# Gender differences in climate change denial in Sweden: the role of threatened masculinity

**DOI:** 10.3389/fpsyg.2024.1450230

**Published:** 2024-12-11

**Authors:** Amanda Remsö, Hanna Bäck, Emma Aurora Renström

**Affiliations:** ^1^Department of Psychology, Kristianstad University, Kristianstad, Sweden; ^2^Department of Political Science, Lund University, Lund, Sweden

**Keywords:** belief in a sexism shift, climate change denial, environmental attitudes, gender identity, threatened masculinity

## Abstract

Previous research in the Western world shows that men are in general more likely than women to deny human-induced climate change or certain aspects of it. We hypothesize that threatened masculinity contributes to such gender differences in Sweden. Threatened masculinity refers to the perception that a man’s masculinity is being challenged, undermined, or devalued, often due to societal changes that advance women’s rights. Given that environmental care and concern are typically associated with femininity, men who perceive that masculinity is threatened may be more likely to deny climate change to restore a sense of masculinity. Across three cross-sectional online surveys with representative samples of Swedish adults (total N = 2,476), men were more likely to deny climate change than women. Threatened masculinity—measured by belief in a shift in sexism and belongingness with men’s rights activists—predicted climate change denial. In line with our hypothesis, belief in a sexism shift and, to a lesser extent, belongingness with men’s rights activists mediated from gender to climate change denial. Hence threatened masculinity contributes to a higher tendency among men compared to women to deny climate change in these samples. This research adds to the understanding of gender gaps in environmental attitudes found in many Western countries and highlights climate change denial as a potential correlate of the growing gender-related polarization observed in these contexts.

## 1 Introduction

While there is now a near-universal consensus in the peer-reviewed scientific literature that human activity is driving climate change (Lynas et al., 2021), some individuals deny this reality or certain aspects of it. Previous research from mainly North America and Western European countries has shown that men as a group compared to women as a group, are more likely to deny the reality of human-induced climate change, less likely to worry about it, and less likely to support climate mitigation policies and behaviors (Agneman et al., 2024; Bush and Clayton, 2023; Gregersen et al., 2020; Hornsey et al., 2016; McCright and Dunlap, 2011, 2013; Poortinga et al., 2019). The present research examines whether perceptions of threatened masculinity contribute to a higher tendency among men compared to women to deny climate change in three representative samples of Swedish citizens.

Empirical evidence across cultures (Bosson et al., 2021) suggests that for many men, manhood is experienced as a precarious identity that requires constant validation through the performance of masculinity, such as enacting masculine behaviors (Bosson and Vandello, 2011; Vandello et al., 2008). Because manhood is not a stable, inherent trait, it can be threatened (Mesler et al., 2022), for example, from societal changes where women gain more rights (Kimmel, 2017; Renström and Bäck, 2024a). In several Western contexts, it is fairly common among the general public to believe that men are now the primary targets of sexism and that the advancement of women’s rights in progressive countries has come at the expense of men’s rights (Ging, 2019; Off et al., 2022; Zehnter et al., 2021). When manhood is threatened, some men respond with hypermasculine behaviors by engaging in stereotypical masculine actions to restore their sense of manhood (Vandello and Bosson, 2013).

Previous research has shown that there is a close relationship between the environment and gender stereotypes (Stoddart and Tindall, 2011), where environmental concern is viewed as a feminine trait (Brough et al., 2016; Swim and Geiger, 2018), while disengagement from environmental issues aligns with traditional notions of masculinity (Stanley et al., 2023; Swim and Geiger, 2018). Denying climate change or certain aspects could be a form of hypermasculinity—an exaggerated expression of masculine traits—to reaffirm a sense of masculinity that is perceived to be under threat. By rejecting what is culturally perceived as feminine and unmanly, some men may attempt to restore a threatened masculinity (Hunt et al., 2016; Renström and Bäck, 2024a; Vandello et al., 2008).

We hypothesize that threatened masculinity contributes to higher climate change denial among men compared to women. Across three representative samples of Swedish adults (total *N* = 2,476), men were more likely to deny climate change than women, and measures of threatened masculinity contribute to a higher tendency among men compared to women to deny climate change in Sweden.

This research adds to the literature on gender differences in environmental attitudes in Western countries by focusing on the contribution of threatened masculinity. More broadly, this research contributes to the understanding of the ideological predictors of climate change denial, which can provide insights for developing targeted climate change communication strategies (Hornsey and Fielding, 2017). Importantly, we also account for previously well-established predictors of climate change denial, including right-wing ideology and social dominance orientation (e.g., Cipriani et al., 2024; McCright et al., 2016).

## 2 Background and theory

### 2.1 Climate change denial

The majority of people around the world (86% on average) see climate change resulting from human activity as a threat (Vlasceanu et al., 2024), yet there remains a tendency in some parts of the public to deny climate change or certain aspects of it. For example, across 21 countries, 9–31% of the population (22% on average) believe that climate change resulting from human activity is a hoax invented to deceive people (Ibbetson, 2021). Others oppose the extent of the human contribution or the severity of its negative consequences for humans and ecosystems (Ekberg et al., 2022). Hence, climate change denial can manifest in various forms, ranging from outright denial of a changing climate (trend) to denial of the human causes (attribution) and denial of its implications (impact), among others (Dunlap, 2013; Rahmstorf, 2004).

What unites various forms of climate change denial, as well as science denial in general, is the rejection and dismissal of well-established scientific evidence or the scientific method as a whole (Jylhä et al., 2022). Climate change denial, in particular, can impede pro-environmental behaviors and actions, thus threatening climate change mitigation efforts (Gifford, 2011). For example, those who believe in conspiracy theories about climate change tend to have less trust in climate science and are also less willing to accept and participate in actions to reduce greenhouse gas emissions (Biddlestone et al., 2022). Therefore, identifying who denies climate change and the individual-level contributing factors is imperative.

### 2.2 Gender gaps in climate change denial in western countries

McCright and Dunlap (2011) introduced the “conservative white male profile,” also known as the “cool-dude effect,” among climate change deniers. They found that conservative white males in the United States are more likely to deny climate change compared to other demographic groups. This cool dude effect extends beyond the United States, as evidenced by research from Norway showing that 63% of conservative males in a Norwegian sample do not believe in human-induced climate change, compared to 36% among the rest of the sample (Krange et al., 2019). Research from Anglophone countries (Hornsey et al., 2016; McCright and Dunlap, 2013) and Western European countries (Agneman et al., 2024; Gregersen et al., 2020; Poortinga et al., 2019) has shown that men, compared to women, are also less likely to worry about climate change and to support and adhere to mitigation policies and behaviors. There is some evidence from a broader set of countries showing that the gender gap in climate change concern is especially pronounced in countries with greater economic development (Bush and Clayton, 2023).

Meta-analytical evidence has shown that the “conservative part” of the cool dude effect is more diagnostic than the “white male part” (Hornsey et al., 2016). In other words, ideology, more than gender (and ethnicity) itself, seems to be the strongest predictor of climate change perceptions among the public in Western countries. In line with this, politically conservative and right-leaning individuals in these contexts report lower belief in the reality of climate change, less support for mitigation policies, less worry about climate change, lower climate change threat perceptions, and lower trust in climate scientists compared to politically liberal and left-leaning individuals (Gregersen et al., 2020; Hornsey et al., 2016, 2018; McCright et al., 2016; Poortinga et al., 2019; Poushter et al., 2022; Remsö et al., 2024).

Specifically, social dominance orientation (SDO; Pratto et al., 1994), which is rooted in social dominance theory (Sidanius and Pratto, 1999), correlates with climate change denial in research with Western European countries and the United States (Cipriani et al., 2024; Hornsey, 2021; Häkkinen and Akrami, 2014; Jylhä and Akrami, 2015; Jylhä et al., 2016, 2021; Stanley et al., 2019; Stanley and Wilson, 2019). SDO captures individuals’ acceptance and endorsement of social hierarchies in society and the dominance of superior groups over inferior groups (Pratto et al., 1994). Men and conservatives tend to score higher on SDO compared to women and liberals (Duckitt and Sibley, 2009; Jost et al., 2003; Pratto et al., 1994). Consistent with this, SDO has been found to mediate the path from both political conservatism and male gender in predicting climate change denial in Brazilian and Swedish samples (Jylhä et al., 2016).

More recent studies from Western countries have also shown that anti-feminist, misogynistic, and sexist attitudes toward women are positively correlated with climate change denial (Benegal, 2018; Jylhä and Hellmer, 2020; Kaul and Buchanan, 2023). Importantly, some studies using Swedish and American samples show that the effect of SDO on climate change denial becomes statistically non-significant when simultaneously including such attitudes (Jylhä et al., 2020; Nicol et al., 2021), indicating that sexist attitudes are closely connected to climate change denial.

### 2.3 Previous research on threatened masculinity

The current research examines whether *threatened masculinity* mediates the difference between men and women in climate change denial in Sweden. To this end, we build on work that has observed an increasing gender-related polarization in many Western societies in recent decades. In particular, there has been a growing emphasis on portraying men rather than women as the primary victims of sexism (Zehnter et al., 2021).

Recently, Zehnter et al. (2021) developed a scale, *belief in a sexism shift* (BSS), to capture the belief that the target of sexism has now shifted from women to men. They argue that while BSS reflects a form of sexism toward women and shares some similarities with more traditional forms of sexism (e.g., Swim et al., 1995), it also has some crucial distinctions. Specifically, unlike most other forms of sexism, BSS operates in a subtler, more socially acceptable manner because its outward focus is on promoting equality for men. Hence, this type of sexism emphasizes male victimhood in a way that masks the underlying hostility and paternalism toward women, making it less overtly anti-female.

This belief, or ideology, centers around the idea that the advancement of women’s rights in progressive countries during recent decades has come at the expense of men’s rights (“the end of men and the rise of women”; Rosin, 2020). In other words, gender equality is seen as a zero-sum game, wherein gains in equality for one gender are perceived as losses for another (Kehn and Ruthig, 2013). Societal changes and progress, where women and minority groups are gaining rights, have been described as one of the root causes of radicalization among some men (Kimmel, 2017). Overall, perceiving one’s group to be discriminated against is related to defending one’s identity and becoming more radical in one’s views (Knapton et al., 2022; Kruglanski et al., 2019).

Individuals and groups that subscribe to the ideas of a sexism shift often refer to themselves and/or are referred to by others as *men’s rights activists* (MRA), as these individuals and groups focus on addressing issues that disproportionately affect men (Ging, 2019). Popularly known as the manosphere, these interest groups primarily operate online, and while some parts of the manosphere have been associated with extremism and occasionally violent misogyny, such as the incel movement (i.e., involuntary celibacy; Czerwinsky, 2024), the belief that sexism has shifted from women to men seems to be fairly common among the general public in much of the Western world.

For instance, approximately two-thirds of American men in 2016 reported that they were facing at least some gender discrimination (ANES, 2016), and 14% of American men, along with 5% of women, think that it is now easier to be a woman than a man (Horowitz et al., 2017). In the United States, while men perceived a decline in anti-women discrimination across six decades, they perceived an increase in anti-men discrimination during the same period (Kehn and Ruthig, 2013). Conservative men in the United States were most likely to report that anti-man bias now equals or exceeds anti-woman bias (Bosson et al., 2012).

Evidence from 27 EU countries shows that younger men, in particular, view women’s progress as a threat to men’s opportunities (Off et al., 2022). More than half (55%) of British men believe that the struggle for women’s rights has gone so far that men are now being discriminated against, an opinion shared by 41% of women (The Global Institute for Women’s Leadership, 2023). About 20% of Danish men and 2% of women between the ages of 18 and 34 believe that gender equality has gone too far (Shamshiri-Petersen et al., 2024). In Sweden, 14% of men and 6% of women believe that gender equality has gone too far in many areas (Tjänstemännens Centralorganisation, 2023).

### 2.4 Threatened masculinity as a mediator between gender and climate change denial

To build our argument on threatened masculinity as a mediator between gender and climate change denial, we draw on previous work on manhood and masculinity. While often used interchangeably in the literature, manhood is about achieving and maintaining the social identity of a man, often through proving one’s masculinity, which is about the behaviors and qualities linked to being male in a specific culture. For instance, proving one’s masculinity could be achieved through acts of aggression or violence (Willer et al., 2013).

Manhood has been described as a precarious identity and something that needs to be constantly proven and maintained through the performance of masculinity (Bosson and Vandello, 2011; Vandello et al., 2008). Notions of precarious manhood are recognized across cultures (Bosson et al., 2021).

According to this view, manhood is not a stable, inherent trait, and consequently, men tend to experience gender identity threats more easily than women (Mesler et al., 2022).

One example of a gender identity threat is societal changes where women gain more rights (Kimmel, 2017; Renström and Bäck, 2024a). The theory of masculine overcompensation (Bosson et al., 2009; Willer et al., 2013) suggests that when manhood is threatened, men tend to react with hypermasculine behaviors and feel compelled to engage in stereotypically manly deeds in order to restore their sense of masculinity (Vandello and Bosson, 2013). For instance, men exposed to masculinity threats in the United States have been found to be more likely to support war, express homophobic attitudes, be interested in purchasing an SUV, and report a stronger attachment to meat (Nakagawa and Hart, 2019; Willer et al., 2013).

There is a close relationship between the environment and gender stereotypes (Stoddart and Tindall, 2011). In many cultures around the world, masculinity is stereotypically associated with agency, characterized by traits such as decisiveness, dominance, and achievement orientation, while femininity is stereotypically associated with communal traits such as empathy, nurturing, and warmth (e.g., Glick and Fiske, 1996). Accordingly, men are expected to be agentic, and women are expected to be communal (Koenig, 2018). This also seems to be how men and women view themselves; evidence from 62 countries shows that women view themselves higher in communion than men and that men view themselves higher in agency than women (Kosakowska-Berezecka et al., 2023).

These gender stereotypes and self-views link femininity, but not masculinity, to environmental care and concern (Stoddart and Tindall, 2011; Rome, 2006). Previous research from mainly Western world countries demonstrates the gendered nature of climate change and environmental issues in general; both women and men associate nature with women rather than men (Liu et al., 2019), and environmental care in particular is perceived as a feminine pursuit (Brough et al., 2016). Individuals have been shown to assume that those who are concerned, alarmed, or cautious about climate change are more likely to be women, while dismissiveness, doubtfulness, and disengagement about climate change are more often perceived to be men (Swim and Geiger, 2018). Men tend to prefer climate change policies that emphasize science, business, and leadership (i.e., agency) over those that focus on justice and caring for others (i.e., communality; Swim et al., 2018). Women seem to favor climate policies that benefit future generations more than men do (Agneman et al., 2024). Additionally, men who strongly identify with masculinity are more likely to consume more meat and are less open to adopting a vegetarian diet (Rosenfeld and Tomiyama, 2021; Stanley et al., 2023).

One key aspect of masculinity in Western culture is the rejection and distancing from anything deemed feminine and unmanly (Hunt et al., 2016). As a result, men who perceive that masculinity is threatened by women’s societal progress may be more likely to deny climate change in order to restore a sense of masculinity (Bosson et al., 2009; Renström and Bäck, 2024a; Vandello et al., 2008; Vandello and Bosson, 2013; Willer et al., 2013). That is, denying climate change or downplaying its significance may align more with stereotypically and/or self-viewed masculine traits, as it may signify a disregard for emotions and concerns perceived as feminine, such as empathy and environmental sensitivity.

**Figure 1:**
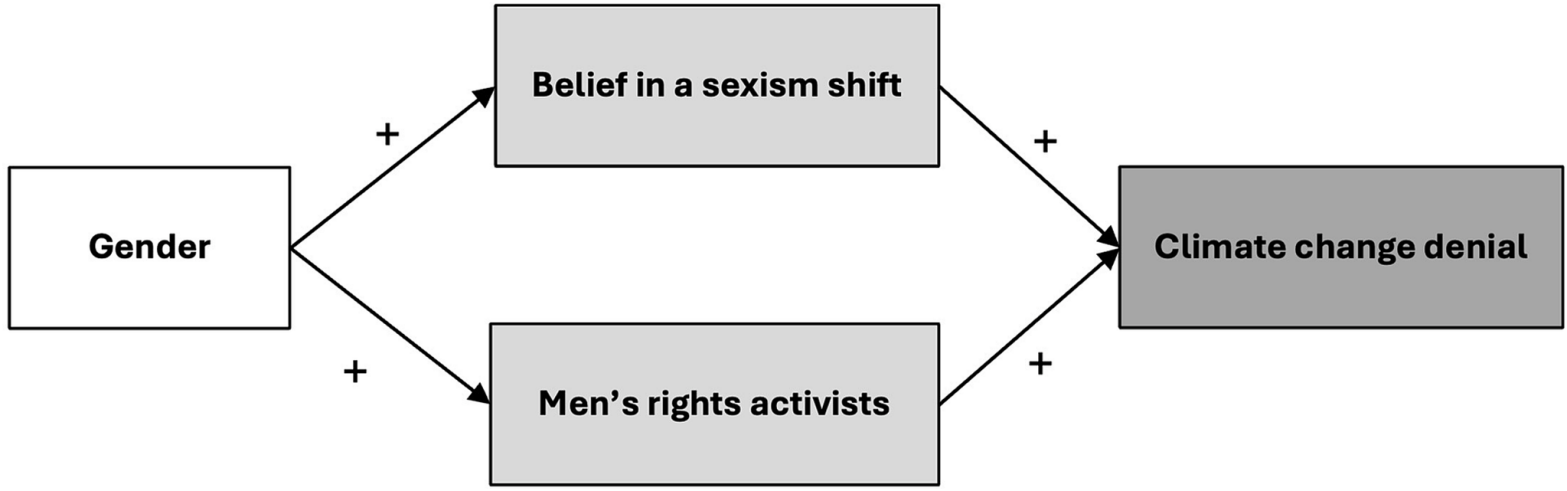
Conceptual mediation model.

However, self-identifying as male will not necessarily be associated with a higher tendency to deny climate change. Rather, we expect that threatened masculinity is associated with a higher tendency to deny climate change and that this perception is more prevalent among men. Based on these arguments, we hypothesize that *threatened masculinity contributes to a higher tendency among men compared to women to deny climate change in Sweden.* Threatened masculinity is operationalized as belief in a sexism shift and belongingness with the group of men’s rights activists. This conceptual mediation model is visualized in Figure 1.

## 3 Methods and data

### 3.1 A case study of gender differences in climate change denial in Sweden

We test the hypothesis in three Swedish samples. Sweden is an ideal setting for this research given the current sociopolitical dynamics surrounding both climate change and gender equality in Sweden. Approximately 2% of the Swedish population consider it entirely false to claim that climate change is mainly caused by human activity (Axelsson and Jönsson, 2023). When broken down by gender, 3% of men and 1% of women share this view. While public opinion on climate issues in Sweden has remained stable since the 1980s, recently, it has become increasingly connected to political partisanship, and gaps between the political left and right have widened (Jönsson, 2022). Sweden mirrors patterns observed in other Western European countries, such that right-wing populist partisans report stronger skepticism toward climate change and lower levels of concern compared to other partisans (Axelsson and Jönsson, 2023; Fisher et al., 2022; Jönsson, 2022; Poushter et al., 2022).

The populist right-wing party, the Sweden Democrats, has increased in support and in the last national election they were the second largest party in Sweden (Valmyndigheten, 2023).

When it comes to gender equality, there seems to be a growing tension in Sweden, which, according to our argument, can be associated with climate change denial. According to the 2024 Global Gender Gap Index, Sweden is one of the most gender-equal countries in the world (World Economic Forum, 2024). Nevertheless, data from the World Value Survey shows that Sweden out of 31 countries has had the greatest deterioration in pro-equality attitudes in areas of politics, economics, education, and psychical integrity between the years of 2005 and 2014 (United Nations Development Programme, 2020).

Analyses of online platforms demonstrate a strong backlash against gender equality in the Nordic countries, wherein self-appointed men’s rights activists frame women’s rights and feminism as a threat to society that must be fought (Centre for Digital Youth Care, 2020). Sweden also has the highest numbers of active users on misogynistic online forums *per capita* in comparison to nine other countries (Fernquist et al., 2020), and Swedish men have been found to score relatively high on the belief that men are the most discriminated group (Renström and Bäck, 2024). Research further indicates strong gender stereotypes in Sweden, with women expected to be more communal than men and men expected to be more agentic than women (Renström, 2024). At the same time, Swedish citizens in general view their society as highly gender egalitarian (Gender Equality Agency, 2022).

### 3.2 Participants and procedure

We report the results of three online surveys conducted in Sweden. In all studies, we use the same variables, unless otherwise indicated, and we will therefore report all three studies combined. The surveys were created in the web survey tool Qualtrics and conducted in Swedish. Participants were recruited by the Swedish survey company *Enkätfabriken,* and the data was collected in January and February 2024.^1^ Participants were invited to take part in a survey about sociopolitical issues. Participants were informed about ethical considerations, including voluntary participation, the right to withdraw, and data handling. Participants provided informed consent by agreeing to participate based on this information by ticking a designated box.

A total of 2,476 participants completed the surveys across the three studies. In Study 1,304 participants finished the survey [*M*_age_ = 47.34, *SD*_age_ = 19.97, range _age_ (18–95); 53.0% women, 45.7% men, 1.3% missing; 45.1% with college or university education]. In Study 2, 1,100 participants took part [*M*_age_ = 49.2, *SD*_age_ = 18.0, range_age_ (18–92); 49.5% women, 48.5% men, 2.0% missing; 42.3% with college or university education]. Finally, in Study 3, 1,072 participants took part in this survey [*M*_age_ = 47.54, *SD*_age_ = 18.00, range _age_ (18–95); 50.9% women, 47.2% men, 1.9% missing; 0.43.2% with college or university education].

### 3.3 Measures

The outcome variable, *climate change denial*, was measured by using three items adapted from a longer scale by Häkkinen and Akrami (2014). The items, for example, “It is unclear if the Earth’s climate is changing,” have responses on a 7-point scale ranging from 1 (*strongly disagree*) to 7 (*strongly agree*). Each individual item taps into various aspects of climate change denial, including denial that climate change is happening (trend), denial of the human cause (attribution), and denial of the large-scale consequences of climate change (impact). These three items were combined into a mean index. See Table 1 for Cronbach’s alpha for all indexes.

**Table 1:** Means, standard deviations, Cronbach’s alpha, and correlations between all variables, studies 1–3.

Study 1	*M* (*SD*)	*α*	Age	Gender	Education	Ideology	SDO	BSS	MRA
Gender			−0.03						
Education	4.16 (1.37)		0.03	−0.02					
Ideology	3.96 (1.76)		−0.02	0.17^***^	−0.01				
SDO^1^	2.93 (1.31)	0.72	−0.09	0.21^***^	−0.13^*^	0.30^***^			
BSS^2^	3.51 (1.61)	0.86	−0.18^***^	0.38^***^	−0.03	0.41^***^	0.54^***^		
MRA^3^	2.98 (1.68)		−0.12^*^	0.15^**^	−0.10	0.14^**^	0.24^***^	0.44^***^	
CC denial^4^	3.01 (1.39)	0.64	−0.16^**^	0.19^***^	−0.22^***^	0.27^***^	0.44^***^	0.53^***^	0.37^***^

Study 2	*M* (*SD*)	*α*	Age	Gender	Education	Ideology	SDO	BSS	MRM
Gender			0.00						
Education	4.16 (1.33)		0.06*	−0.07*					
Ideology	4.15 (1.69)		−0.05	0.08**	−0.01				
SDO^1^	2.99 (1.20)	0.69	−0.16***	0.10***	−0.10***	0.37***			
BSS^2^	3.40 (1.56)	0.84	−0.24***	0.31***	−0.02	0.29***	0.40***		
MRA^3^	2.96 (1.73)		−0.24***	0.12***	−0.03	0.06*	0.14***	0.37***	
CC denial^4^	2.97 (1.38)	0.68	−0.21***	0.08**	−0.10***	0.33***	0.52***	0.41***	0.22***

Study 3	*M* (*SD*)	*α*	Age	Gender	Education	Ideology	SDO	BSS	MRM
Gender			0.04						
Education	4.21 (1.33)		0.07*	−0.07*					
Ideology	4.12 (1.75)		−0.02	0.07*	−0.08**				
SDO^1^	2.94 (1.24)	0.73	−0.17***	0.20***	−0.15***	0.38***			
BSS^2^	3.27 (1.24)	0.84	−0.18***	0.39***	−0.10***	0.23***	0.43***		
MRA^3^	2.91 (1.65)		−0.16***	0.17***	−0.02	0.07*	0.19***	0.33***	
CC denial^4^	2.94 (1.38)	0.67	−0.20***	0.15***	−0.18***	0.28***	0.50***	0.42***	0.29***

*p < 0.05, **p < 0.01, and ***p < 0.001. Gender is dummy coded (men = 1, women = 0). ^1^Social dominance orientation. ^2^Belief in a sexism shift. ^3^Men’s rights activists. ^4^Climate change denial.

The focal predictor variable and proposed mediator, *threatened masculinity*, was operationalized by using two separate measurements, which are included as two variables in the analyses: belief in a sexism shift (BSS) and belongingness with the group of men’s rights activists (MRA). The purpose of using both variables was to account for both the attitudinal and group dimensions of threatened masculinity, as research demonstrates that science denial can be related both to individual attitudes and the social groups an individual identifies with (see, e.g., Hornsey and Fielding, 2017; Rutjens and Hornsey, 2024).

To measure *belief in a sexism shift* (BSS), we used items from a longer scale from Zehnter et al. (2021). This scale captures the extent to which participants agree with statements portraying men rather than women as the primary victims of sexism in contemporary societies. An example item is: “In Sweden, discrimination against men is on the rise,” with responses on a 7-point scale ranging from 1 (*strongly disagree*) to 7 (*strongly agree*). We used eight of the original 15 items in Study 1 and four of the items in Studies 2 and 3. The items were combined into a mean index.

The second measurement to capture threatened masculinity, *men’s right activist belongingness* (MRA), captures the extent to which participants experience a sense of belonging or affinity with groups that advocate for what they perceive as rights and issues affecting men, often in response to what they see as disparities or disadvantages faced by men (Ging, 2019). The item read: “People sometimes talk about different groups in society that people identify with. To what extent would you say you feel close to the group of men’s rights activists?.” This item was rated on a scale from 1 (*not close at all*) to 7 (*very close*).

To measure participant *self- identified gender identity*, the following response options were listed: *female*, *male*, *other*, and *prefer not to say*. Since our hypothesis focuses on differences between men and women regarding climate change denial, responses of “other” and “prefer not to say” were coded as missing values for the analyses.

Control variables included *social dominance orientation* (SDO), which captures individuals’ desire to maintain and establish hierarchically structured intergroup relations (Pratto et al., 1994). This variable was measured using the four-item scale by Pratto et al. (2013), more recently adapted and validated by Aichholzer and Lechner (2021), for example, “Superior societal groups should dominate inferior groups,” and responses were made on a 7-point scale ranging from 1 (*strongly disagree*) to 7 (*strongly agree*). The items were combined into a mean index.

Additional control variables were age, education, and left–right ideology. *Age* was measured in years and treated as a continuous variable. *Education* was measured on a 7-point scale ranging from 1 (*less than high school*) to 7 (*doctoral degree*); thus, higher scores represent higher education and were treated as a continuous variable. The item measuring *ideology* read: “Sometimes political opinions are described on a scale from left to right. Where would you place yourself?” with responses from 1 (*clearly to the left*) to 7 (*clearly to the right*). Thus, higher scores represent a stronger right-leaning ideology. See Supplementary materials for further details on all items.

## 4 Results

### 4.1 Descriptive results

First, we present descriptive statistics and zero-order correlations between all variables in Table 1. As can be seen, men (compared to women) reported higher climate change denial in all three studies. BSS, reflecting the belief that men are now the primary targets of sexism due to women’s societal advancement, and MRA, indicating a stronger sense of belonging with groups advocating for men’s rights, were also positively correlated with climate change denial in all studies. Additionally, in all studies, SDO showed positive correlations with denial, as did stronger right-wing ideology, older age, and lower education. Finally, BSS and MRA were moderately positively correlated in all three studies.

Next, we tested the mean differences in climate change denial between men and women using independent *t*-tests. The results, presented in Table 2, indicate that across all three studies, men reported higher climate change denial than women, with small to moderate effect sizes.

**Table 2:** Climate change denial mean differences and *t*-tests between men and women, Studies 1–3.

		M (*SD*)	*t* (df)	*p*-value	Cohen’s *d*
Study 1	Men	3.29 (1.41)	3.34 (296.00)	<0.001	0.39
	Women	2.76 (1.34)			
Study 2	Men	3.09 (1.42)	2.71 (1015.00)	<0.01	0.17
	Women	2.85 (1.33)			
Study 3	Men	3.15 (1.41)	4.92 (994.00)	<0.001	0.31
	Women	2.73 (1.31)			

### 4.2 Predicting threatened masculinity

Before turning to the main analyses, we first present results on the proposed mediator between gender and climate change denial: threatened masculinity, as measured by BSS and MRA. We tested the mean differences between men and women using independent *t*-tests. The results indicate that across all three studies, men scored higher on both BSS and MRA compared to women, with small, moderate, and large effect sizes (see Supplementary materials).

To determine whether men’s tendency compared to women to report higher scores on threatened masculinity persists when demographic and ideological variables are taken into account, we conducted multiple linear regression analyses. Each outcome variable, BSS and MRA, was analyzed separately across all three studies. The predictors included age, education, gender, ideology (left–right), and SDO.

The results, presented in Table 3, demonstrate that the positive association between gender and threatened masculinity remains for both outcomes when controlling for the other variables (except in Study 1, predicting MRA). Older individuals report higher scores on threatened masculinity (except in Study 1, predicting MRA). In all studies, a stronger right-wing ideology predicted BSS but not MRA. SDO predicted higher threatened masculinity on both outcomes across all three studies. Finally, education level did not predict either outcome in either study.

**Table 3:** Multiple linear regression models predicting threatened masculinity, studies 1–3.

	Belief in a sexism shift			Men’s rights activists		
	Study 1	Study 2	Study 3	Study 1	Study 2	Study 3
	*B (SE)*	*B (SE)*	*B (SE)*	*B (SE)*	*B (SE)*	*B (SE)*
Age	−0.01 (0.00) ***	−0.02 (0.00) ***	−0.01 (0.00) ***	−0.01 (0.01)	−0.02 (0.00) ***	−0.01 (0.00) ***
Gender	0.85 (0.15) ***	0.86 (0.09) ***	1.00 (0.08) ***	0.29 (0.20)	0.41 (0.11) ***	0.45 (0.11) ***
Education	0.06 (0.05)	0.04 (0.03)	−0.02 (0.03)	−0.07 (0.07)	−0.02 (0.04)	0.01 (0.04)
Ideology	0.21 (0.04) ***	0.13 (0.03) ***	0.08 (0.03) ***	0.07 (0.06)	0.00 (0.03)	0.00 (0.03)
SDO^1^	0.52 (0.06) ***	0.39 (0.04) ***	0.38 (0.04) ***	0.25 (0.08) ***	0.13 (0.05) *	0.20 (0.05) ***
*R^2^_adj_*	0.45***	0.28***	0.31***	0.07***	0.08***	0.07***

*p < 0.05, **p < 0.01, and ***p < 0.001. Gender is dummy coded (men = 1, women = 0). ^1^Social dominance orientation.

### 4.3 Mediation analyses

In this section, we test the full hypothesis that threatened masculinity contributes to a higher tendency among men compared to women to deny climate change in Sweden. To test this, we conducted mediation analyses.^2^ We use mediation analyses to probe whether threatened masculinity accounts for some of the statistical relationship between gender and climate change denial without claiming that this is a cause-and-effect chain. These analyses used 5,000 bootstrapped iterations to generate confidence intervals for each path in the models. Climate change denial was the outcome variable, with gender as the main predictor (coded as a dummy variable: 1 = men, 0 = women). BSS and MRA were both included as mediators. The control variables were age, education, ideology (left/right), and SDO.

Table 4 displays the bootstrapped coefficients for indirect paths (mediated), direct paths (gender to denial accounting for mediators), and all predictor variables, with and without the mediators.

**Table 4:** Mediation analyses predicting climate change denial, studies 1–3.

Mediation	Study 1		Study 2		Study 3	
	*B (SE)*	95% CI	*B (SE)*	95% CI	*B (SE)*	95% CI
Indirect BSS^1^	0.22 (0.07)	0.10, 0.36	0.15 (0.03)	0.09, 0.22	0.18 (0.04)	0.11, 0.26
Indirect MRA^2^	0.04 (0.03)	−0.01, 0.12	0.03 (0.01)	0.01, 0.06	0.05 (0.02)	0.02, 0.09
Direct Gender	−0.03 (0.15)	−0.32, 26	−0.11 (0.08)	−0.26, 0.04	−0.08 (0.08)	−0.24, 0.08
With mediators						
Age	−0.01 (0.00)	−0.01, 0.00	−0.01 (0.00)	−0.01, 0.00	−0.01 (0.00)	−0.01, 0.00
Education	−0.16 (0.05)	−0.26, −0.06	−0.05 (0.03)	−0.10, 0.00	−0.09 (0.03)	−0.15, −0.04
Ideology	0.05 (0.04)	−0.04, 0.13	0.10 (0.02)	0.06, 0.15	0.08 (0.02)	0.03, 0.12
SDO^3^	0.22 (0.06)	0.10, 0.34	0.43 (0.04)	0.36, 0.50	0.36 (0.04)	0.29, 0.43
BSS	0.26 (0.06)	0.14, 0.37	0.17 (0.03)	0.11, 0.23	0.18 (0.03)	0.12, 0.24
MRA	0.12 (0.04)	0.04, 0.21	0.06 (0.02)	0.02, 0.11	0.12 (0.02)	0.07, 0.17
*R* ^2^_adj_	0.37***		0.34***		0.33***	
Without mediators						
Age	−0.01 (0.00)	−0.01, 0.00	−0.01 (0.00)	−0.01, −0.01	−0.01 (0.00)	−0.01, −0.01
Gender	0.17 (0.08)	0.02, 0.32	0.06 (0.08)	−0.08, 0.21	0.17 (0.08)	0.01, 0.33
Education	−0.10 (0.03)	−0.16, −0.05	−0.04 (0.03)	−0.10, 0.01	−0.10 (0.03)	−0.16, −0.05
Ideology	0.09 (0.03)	0.04, 0.14	0.13 (0.03)	0.08, 0.17	0.09 (0.03)	0.04, 0.14
SDO	0.45 (0.04)	0.37, 0.52	0.50 (0.04)	0.43, 0.58	0.45 (0.04)	0.37, 0.52
*R^2^_adj_*	0.28***		0.31***		0.28***	

***p < 0.001. Gender is dummy coded (men = 1, women = 0). ^1^Belief in a sexism shift. ^2^Men’s rights activists. ^3^Social dominance orientation. B = bootstrapped unstandardized coefficients based on 5,000 iterations. SE, standard error of bootstap estimate; 95% CI, lower limit and upper limit 95% confidence interval.

In Study 1, BSS, but not MRA, mediated the relationship between gender and climate change denial. When the mediators were included, gender had no direct association with climate change denial. Without the mediators, however, gender predicted higher denial. In Study 2, BSS again mediated the relationship between gender and climate change denial, as did MRA, but to a lesser extent. Gender did not predict climate change denial with or without the mediators. In Study 3, both BSS and MRA mediated the relationship between gender and climate change denial, with MRA again to a lesser extent. Gender did not predict climate change denial when mediators were included, but the relationship was weakly supported without them.

Finally, when accounting for the mediators, individuals with higher SDO scores reported higher denial across all studies. In Studies 2 and 3, a stronger right-wing ideology was also associated with higher denial. Lower education levels predicted higher denial in Studies 1 and 3 but not in Study 2, while age did not predict denial in either study.

In sum, these analyses suggest that BSS, and to a lesser extent, MRA, contributes to the relationship between gender and climate change denial. Gender did not predict climate change denial in the presence of the mediators but did so in Studies 1 and 3 without them. Thus, these results support the hypothesis that threatened masculinity contributes to higher climate change denial among men compared to women across three Swedish samples. Specifically, men who perceive that men, rather than women, are now the primary victims of sexism due to women’s societal progress also report higher scores on climate change denial. Additionally, men who experience belongingness with men’s rights activists, which are groups focused on addressing issues perceived to disproportionately affect men, also show a somewhat higher tendency to deny climate change. These results were found while controlling for age, education, left/right ideology, and SDO.

## 5 Discussion

Despite the near-unanimous scientific consensus that human-induced climate change is real and poses substantial threats to nature and global health (IPCC, 2023; Lynas et al., 2021), various forms of climate change denial persist within segments of the public. In three online surveys of representative samples of Swedish adults (total *N* = 2,476), we found, consistent with earlier research from Western countries (Hornsey et al., 2016; McCright and Dunlap, 2011), that men reported higher climate change denial than women. We hypothesized that threatened masculinity contributed to a higher tendency among men compared to women to deny climate change in Sweden. Threatened masculinity was operationalized as belief in a sexism shift and a sense of belongingness with the groups of men’s rights activists. The findings showed that belief in a sexism shift mediated the relationship between gender and climate change denial across all three studies. The sense of belonging to men’s rights groups mediated this relationship in Studies 2 and 3, and to a lesser extent than belief in a sexism shift.

Overall, these findings contribute to the literature on some of the contributing factors of men being more likely than women to deny climate change in Western countries. Given that the relationship between gender and climate change denial disappeared once threatened masculinity was accounted for, these findings support the notion that ideology, more than gender in itself, contributes to these differences between men and women (Hornsey et al., 2016). In other words, one contributing factor as to why men are more likely to deny climate change compared to women seems to be the fact that men, on average, hold different ideological views, particularly with regards to gender equality and sexism. This also aligns with previous research showing that anti-female sexism is associated with climate change denial in North America and Western Europe (Benegal, 2018; Jylhä and Hellmer, 2020; Jylhä et al., 2020; Kaul and Buchanan, 2023; Nicol et al., 2021).

Notably, we controlled for previously well-established predictors of climate change denial, including right-wing ideology (e.g., Hornsey et al., 2016; McCright et al., 2016) and social dominance orientation, which has also been found to mediate the path from male gender to climate change denial (Jylhä et al., 2016). The current findings extend previous research to show that threatened masculinity, as indexed by belief in a sexism shift (BSS; Zehnter et al., 2021) and belongingness with men’s rights activists (MRA; Ging, 2019), predicts denial beyond SDO and right-wing ideology. Hence, these measures of threatened masculinity are not merely additional indicators of right-wing or broader status-legitimizing ideologies but reflect a distinct contribution to climate change denial.

Interestingly, belief in a sexism shift and belongingness with men’s rights activists showed only a moderate zero-order correlation across all three studies, indicating that while there are some commonalities between attitudes related to threatened masculinity and group belongingness, they are distinct constructs. Yet the fact that the predictive power of BSS and MRA did not overlap but instead contributed unique and relatively strong varience predicting climate change denial suggests that both individuals’ attitudes and group belongingness in the context of threatened masculinity are related to denial (see, e.g., Hornsey and Fielding, 2017; Rutjens and Hornsey, 2024). Of course, the current research is correlational and does not imply causality, meaning that individuals who deny climate change may also be more likely to adopt a sense of threatened masculinity, whether as attitudes or group belongingness.

However, we anchor these findings in the literature, which describes manhood as something that needs to be consistently proven and maintained through the performance of traditional masculine traits (Bosson and Vandello, 2011; Vandello et al., 2008). Because environmental care and concern are perceived as stereotypically feminine traits (e.g., Brough et al., 2016; Stoddart and Tindall, 2011), individuals who perceive a sense of threatened masculinity, which is more common among men in our studies, for example, due to women’s societal progress, may deny climate change to reject what they perceive as feminine and unmanly, thus restoring a sense of masculinity; so-called masculine overcompensation (Bosson et al., 2009; Mesler et al., 2022; Vandello and Bosson, 2013; Willer et al., 2013).

### 5.1 Implications

One important implication of this research is that threatened masculinities do not only seem to be linked to radicalization and misogynistic attitudes, as seen in previous studies (Kalish and Kimmel, 2010; Kimmel, 2017; Renström and Bäck, 2024a, 2024), but also to the polarization of other salient sociopolitical issues in Western contexts. This insight is important given that the perceptions of threatened masculinity in Western culture are fairly widespread among the general public (e.g., ANES, 2016; Off et al., 2022; Renström and Bäck, 2024a, 2024), and the manosphere (the online milieu where these ideas largely originate from) has moved from the fringes of the internet to mainstream platforms (Jones et al., 2020; Jane, 2018).

This may be particularly pertinent in Sweden, where both climate change and gender equality have become increasingly polarized and politicized in recent decades (e.g., Jönsson, 2022; Jämställdhetsmyndigheten, 2022; Tjänstemännens Centralorganisation, 2023; Renström, 2024). However, considering that political polarization on climate change extends across Western Europe (Fisher et al., 2022) and is especially pronounced in Anglophone countries like the United States (Hornsey et al., 2018; Smith and Mayer, 2019), and that gender issues have become increasingly polarized across these contexts too (The Global Institute for Women’s Leadership, 2023; United Nations Development Programme, 2020), the current findings from Swedish samples are also likely to replicate in other Western countries.

Furthermore, across all three studies, the mean index of climate change denial was below the midpoint of the scale for both men and women. Although these results do not indicate that climate change denial is “mainstream” in Sweden (see also Oscarsson et al., 2021), we do not dismiss it as insignificant. Science denial is not just a matter of individual beliefs but can pose risks to society by undermining evidence-based policymaking and eroding trust in democratic institutions (e.g., Lewandowsky and Oberauer, 2016, 2021). Even if held by a minority, climate change denial could slow down mitigation efforts (Gifford, 2011). Our findings suggest that addressing climate change denial may require engaging with the stereotypes and expectations of masculinity in societies where women and minority groups are gaining more rights.

Specifically, climate communication strategies could be tailored in ways that do not associate environmental care and concern exclusively with stereotypical femininity while neglecting masculinity (Stoddart and Tindall, 2011; Rome, 2006). Instead, acknowledging the reality of climate change and promoting environmental advocacy can be framed as traits that are equally masculine and feminine. One approach could be to frame climate messaging in ways that resonate with traits traditionally associated with masculinity, such as agency and leadership. For example, highlighting male leaders in environmental movements or by using messages that emphasize assertiveness and initiative (e.g., “Take charge of our planet…”).

### 5.2 Limitations and future research

Some limitations in our research warrant discussion. First, all three samples consisted of Swedish adults aged 18–95, with a nearly equal distribution of men/women, approximately half of the sample with a college or university education, which aligns with national statistics (Statistics Sweden, 2022), suggesting that our findings can generalize to the broader Swedish population. However, as we did not collect data on ethnicity and/or cultural differences, we cannot draw any conclusions about potential differences in that regard. We expect that our findings would be replicated in other Western countries (i.e., primarily countries in North America and Western Europe), as our hypothesis is based on previous research from those contexts. However, we cannot speculate to what extent these results can be generalized to other parts of the world (i.e., countries and regions in Africa, Asia, Latin America, and parts of Eastern Europe). For example, research indicates that ideology tends to have a more substantial predictive power on individuals’ attitudes toward climate change and science in wealthier, more democratic countries and in countries with higher greenhouse gas emissions (Hornsey et al., 2018; Jiang and Wan, 2023; Remsö et al., 2024; Younger-Khan et al., 2024).

Relatedly, assuming that threatened masculinity is (at least partly) a response to women’s societal advancement, we would expect its prevalence to vary depending on the level of gender equality in a given country. Some findings support this, as research shows that men in Sweden report higher mean scores on BSS compared to American men (Renström and Bäck, 2024; Zehnter et al., 2021). Hence, this may be attributed to Sweden being one of the most gender-egalitarian countries in the world (World Economic Forum, 2024). It is not clear whether threatened masculinity is a relevant construct in other, less egalitarian countries.

Second, our operationalization of climate change denial included three items: denial that climate change is happening (trend), denial of the human cause (attribution), and denial of the large-scale consequences of climate change (impact), which we then aggregated into a mean index. This approach has been criticized for oversimplifying the broad spectrum of attempts to undermine the scientific consensus on climate change. Specifically, “climate change denial” has been argued to create a dichotomous view between deniers and non-deniers, which could polarize society and distract focus from the issue itself (Almiron and Moreno, 2022; Bretter and Schulz, 2023). Because of this, alternative terms such as climate “obstructionism” and “delayism” have been proposed (Ekberg et al., 2022) to capture a spectrum of attitudes rather than a clear-cut binary division (Dunlap, 2013).

Furthermore, recent analyses from the Center for Countering Digital Hate (2024) highlight a shift toward what is termed “new denial” on platforms like YouTube over the past 5 years. This new denial centers around claims that the impacts of climate change are beneficial or harmless, that climate solutions will not work, and that climate science and the climate movement are unreliable. Concurrently, “old denial” claims, such as denying the existence of climate change or the human role in climate change, seem to have declined, at least to some degree. Although the index used in our studies encompasses both “old” and “newer” forms of denial, future research can study how threatened masculinity and various strategies to undermine scientific consensuses on climate change are related. Understanding the role of threatened masculinity in the online discourse of climate change denial could be particularly relevant, especially in the context of online communities like the manosphere (Jones et al., 2020; Jane, 2018).

Third, given that all three studies were cross-sectional, we cannot make causal inferences about the direction of the relationship between threatened masculinity and climate change denial. It could be the case that climate change denial might lead individuals to adopt certain attitudes or join specific groups that legitimize and validate their pre-existing beliefs about climate change. For instance, individuals who deny climate change might feel a stronger sense of belonging to men’s rights activist groups if climate change denial is an accepted or even favored attitude within those groups. Additionally, there may be unmeasured confounding variables that contribute to the observed relationship by influencing both threatened masculinity (the mediator) and climate change denial (the outcome). One way future research can further explore the direction of the relationship and potential confounders is to experimentally manipulate masculinity threats. Another approach could be to analyze longitudinal data from social media to examine the alignment between the discourse on climate change denial and threatened masculinity, particularly within communities such as the manosphere (see, e.g., Chen et al., 2021).

Our final suggestion for future research is based on recent cross-country work, demonstrating that men, more than women, tend to be less concerned about climate change when countries are wealthier (Bush and Clayton, 2023). The authors suggest that at the individual level, the perceived costs of climate change mitigation increase more for men than for women as countries develop economically, but they did not fully theorize the origins of individuals’ perceptions about the costs of mitigating climate change. Building on our findings, it could be the case that in wealthier countries, which are most often also more gender egalitarian (World Economic Forum, 2024; The World Bank, 2023), the perceived costs of mitigation increase more for men than for women due to perceived threats to traditional masculinity.

Therefore, in countries where traditional gender roles and norms are less rigid, men may perceive higher costs associated with climate change mitigation compared to women, rooted in the threat perception of losing traditional roles and status associated with masculinity. As such, future multi-country research could investigate the correlation between gender gaps in climate change denial and gender equality across countries, as well as whether that is associated with threatened masculinity.

## 6 Conclusion

In line with previous research, this research demonstrates a gender gap in climate change denial in Sweden, with men being more likely to deny climate change than women. This gap, with men being more likely to deny, was mediated by threatened masculinity, which was operationalized as belief in a sexism shift and a sense of belongingness with men’s rights activists. This study adds to the literature on gender differences in environmental attitudes in Western countries, demonstrating that threatened masculinity contributes to such differences. More broadly, these findings suggest that the increasing polarization of climate and gender issues observed in many Western societies during recent decades may be mutually reinforcing.

## Data Availability

The data are openly available on the Open Science Framework: https://osf.io/zqtc4/.

## Footnotes

^1^All three studies were approved by the Swedish Ethical Review Authority, no.: 2023-07073-02.

^2^We used the PROCESS macro tool for SPSS (Hayes, 2018), model 4.
