# Supplementary material for: Gender differences in climate change denial in Sweden: the role of threatened masculinity
Source: Front Psychol. 2024 Dec 11;15:1450230. doi: 10.3389/fpsyg.2024.1450230 (PMC11673762; doi:10.3389/fpsyg.2024.1450230)
Supplement: Supplementary file 1 [file Data_Sheet_1.PDF]

Supplementary Material

1 Supplementary Tables

Table 1. Mean differences and *t*-tests between men and women for belief in a sexism shift and men's rights activists, Studies 1-3.

	Study 1				Study 2				Study 3			
	Men	Women	*p*-value	Cohen's *d*	Men	Women	*p*-value	Cohen's *d*	Men	Women	*p*-value	Cohen's *d*
	M (*SD*)	*M* (*SD*)			*M* (*SD*)	*M* (*SD*)			*M* (*SD*)	*M* (*SD*)		
BSS[1]	4.18 (1.44)	2.94 (1.54)	<.001	0.83	3.88 (1.52)	2.90 (1.45)	<.001	0.66	3.88 (1.42)	2.70 (1.40)	<.001	0.84
MRA[2]	3.24 (1.64)	2.73 (1.69)	<.01	0.30	3.16 (1.76)	2.75 (1.67)	<.001	0.24	3.22 (1.68)	2.64 (1.57)	<.001	0.35

Note.[1]Beleif in a sexism shift. [2]Men's rights activists.

2 Supplementary Study Materials

Studies 1-3, Survey items.

Age:

"How old are you?" Measured in years.

Gender:

"What is your gender?" Male, female, other, and prefer not to say as response options. The options other and prefer not to say were coded as missing values for the analyses.

Education:

"What is your highest level of education? If you have an ongoing education, you take that one." Options ranged from 1 (*less than high school*) to 7 (*doctoral degree*).

Left-right ideology:

"Sometimes political opinions can be placed on a scale from left to right. Where would you place yourself?" from 1 (c*learly to the left*) *to 7* (c*learly to the right*).

Men's rights activists:

"People sometimes talk about different groups in society that people identify with. To what extent would you say you feel close to the group of men's rights activists?"1 (*not close at all*); 7 (*very close*).

Belief in sexism shift:

1 (*strongly disagree*); 7 (*strongly agree*).

Study 1:

1. All in all, men are well respected in today's society (reverse-coded).
2. It is evident that the media is biased against men.
3. Feminism does not discriminate against men (reverse-coded).
4. If anything, men are more discriminated against than women these days.

Studies 1-3:

5. In today's society, women can say things that men are not allowed to say.
6. In today's society, men are often punished for acting manly.
7. In Sweden, discrimination against men is on the rise.

8. Given women more rights often require taking away men’s rights.

Social dominance orientation:

1 (*strongly disagree*); 7 (*strongly agree*).

1. Superior societal groups should dominate inferior groups.
2. In setting priorities, we must consider all societal groups (reverse-coded).
3. We should not push for equality of societal groups.
4. The equality of societal groups should be our goal (reverse-coded).

Climate change denial:

1 (*strongly disagree*); 7 (*strongly agree*).

1. It is unclear if the Earth's climate is changing.
2. Climate change is primarily caused by human activities (reverse-coded).
3. Climate change will not affect life on Earth in any significant way.
